# A case report of placental mesenchymal dysplasia: A rare case of a genetically normal fetus with severe intrauterine growth restriction

**DOI:** 10.1097/MD.0000000000014554

**Published:** 2019-02-22

**Authors:** Bogdan Doroftei, Sabina Neculai-Valeanu, Gabriela Simionescu, Delia Grab, Natalia Plopa, Emil Anton, Radu Maftei

**Affiliations:** a Department of Mother and Child Medicine, University of Medicine and Pharmacy “Gr. T. Popa” Iasi; b Clinical Hospital “Cuza Voda” Iasi; c Origyn Fertility Center Iasi; d Department of Morphostructural Sciences I, University of Medicine and Pharmacy “Gr. T. Popa”, Iasi, Romania.

**Keywords:** Beckwith–Wiedemann syndrome, intrauterine fetal growth restriction, molar pregnancy, placental mesenchymal dysplasia

## Abstract

**Rationale:**

We report a rare case of a pregnant woman with placental mesenchymal dysplasia (PMD) and intrauterine growth restriction (IUGR) with a genetically normal fetus.

**Patient concerns:**

A 42-year-old woman Gravida I, Para I with pre-existent uncontrolled hypertension and uterine polyfibromatosis present at 30 weeks of gestation for diminished fetal activity during the last 2 days.

**Diagnosis:**

Placental mesenchymal dysplasia associated with intrauterine growth restriction, hypertension, and uterine polyfibromatosis.

**Intervention:**

A live male infant was delivered by emergency caesarean section.

**Outcomes:**

The infant, weighing 700 g, died 4 days after birth due to a massive intracerebral hemorrhage.

**Lessons:**

A careful examination should be done at every ultrasound in case of a fetus with IUGR to exclude some rare cases of placental pathologies. PMD can be a rare cause of IUGR with a genetically normal fetus.

## 1 Introduction

Placental mesenchymal dysplasia (PMD) is a rare benign disorder of the placenta characterized by placentomegaly and grapelike vesicles that can resemble at ultrasound examination with a molar pregnancy.^[1]^ PMD was described initially by Moscoso et al^[2]^ as stem villous hyperplasia with elevated maternal serum alpha-feto-protein and enlarged placentas with ultrasound features that are suggestive of partial mole. This condition may resemble a partial hydatidiform mole, however in contrast to partial mole throphoblastic proliferation is absent, the disorder being characterized by aneurysmal dilatation of vessels on the fetal surface of the placenta with dilated stem villi. Although there are some papers that report an incidence around 0.02% of pregnancies,^[3]^ the true incidence of PMD is unknown because it has been previously reported under a variety of names such as “placentomegaly with massive hydrops of placental stem villi” and “pseudopartial moles.”^[4,5]^ Moreover, placental mesenchymal dysplasia is associated with fetal growth restriction (IUGR) in the majority of the cases and in approximately one quarter of the reported cases with Beckwith–Wiedemann syndrome (BWS)^[4,6]^ or intrauterine fetal demise (IUFD), but can also be associated with normal appearing fetuses.

## 2 Case report

**Figure 1:**
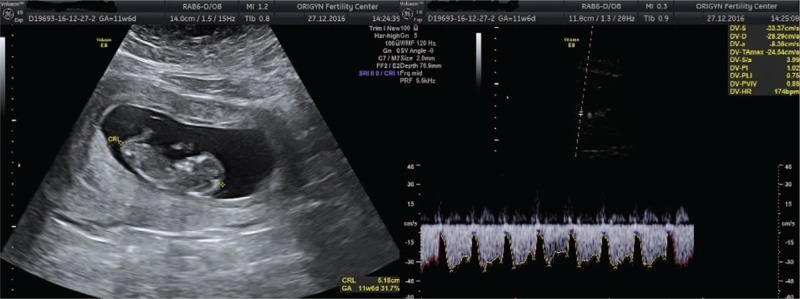
Ultrasound examination at 11 weeks showing a normal appearance of the placenta, normal ductus venosus flow, and CRL corresponding to gestational age. CRL = crown rump length.

We report the case of a 42-year-old woman, Gravida 1, Para 1, that presented to the hospital due to diminished fetal activity in July 2017. Before obtaining this spontaneous pregnancy, the patient was previously diagnosed with a history of almost 10 years of infertility, uterine poly-fibromatosis, hypertension, and obesity grade II. The first ultrasound was performed at 11 weeks and 6 days and revealed a single intrauterine pregnancy. The following parameters were determined during the ultrasound exam: crown rump length (CRL) (51.8 mm), nuchal translucency (1.3 mm), nasal bone present, and ductus venosus flow (Fig. 1). Patient underwent a noninvasive prenatal test and the results were: “low risk”, sex male fetus, and a 4.1% fetal fraction.

**Figure 2:**
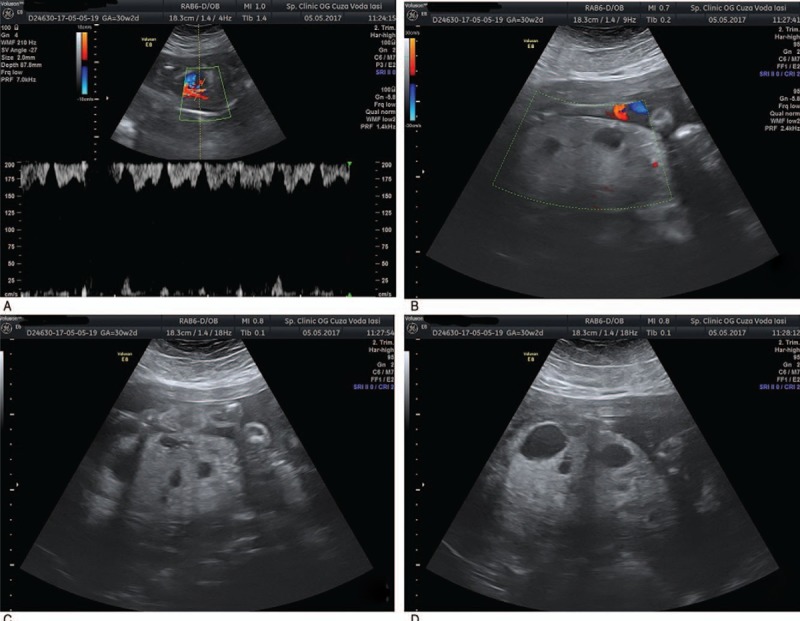
A: ductus venosus with negative a wave; B–D: placentomegaly with multicystic appereance without vascular flow.

Further on, the physical examination of the patient revealed that the patient measured 170 cm in height and 113 kg in weight, having a body mass index (BMI) of 39.1. The blood pressure was 140/90 mmHg thereby the patient was advised to undergo a cardiologic examination in order to change the treatment for the pre-existing hypertension. Unlike the second trimester morphology, which was performed at 21 weeks and had normal results, the ultrasound examination performed at 30 weeks showed severe IUGR (780 g) <2.9 percentile, oligoamnios (amniotic fluid index—AFI: 7), ductus venosus with a negative wave, umbilical artery with reversed diastolic flow and placentomegaly with multicystic appearance without vascular flow (Fig. 2).

Due to these findings, the patient was referred in emergency to the department of obstetrics and gynecology of Cuza Voda Clinical Hospital. At hospital admission, blood pressure was 160/110 mmHg and the medical team initiated treatment with Dopegyt 1tb/6 h and Nifedipine 1tb/12 h. Non-stress test was performed and revealed low reactivity, thereby an amniocentesis was performed and treatment with dexamethasone 6 mg/12 h was initiated. Subsequently, after 48 hours a C-section was performed resulting a male fetus with normal appearance, weighting 700 g, Appearance, Pulse, Grimace, Activity, Respiration score 3. The result from amniocentesis confirmed the absence of genetic anomalies, but the fetus died 4 days later after a massive intracerebral hemorrhage. The milestones related to diagnosis and interventions are depicted in (Table 1).

**Table 1:**
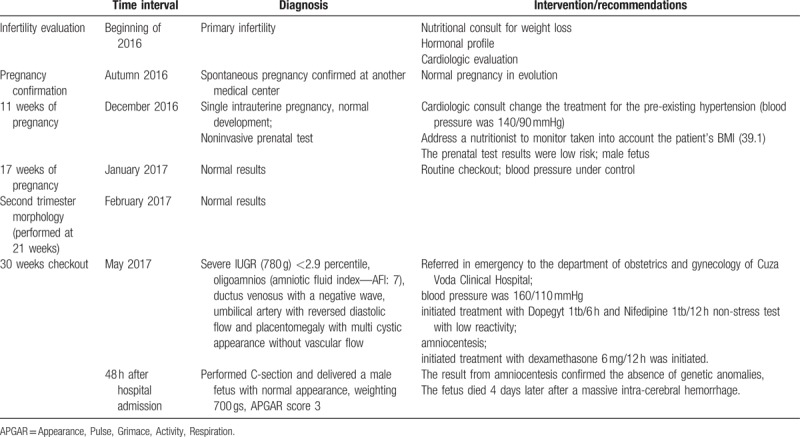
Important milestones related to your diagnosis and interventions.

**Figure 3:**
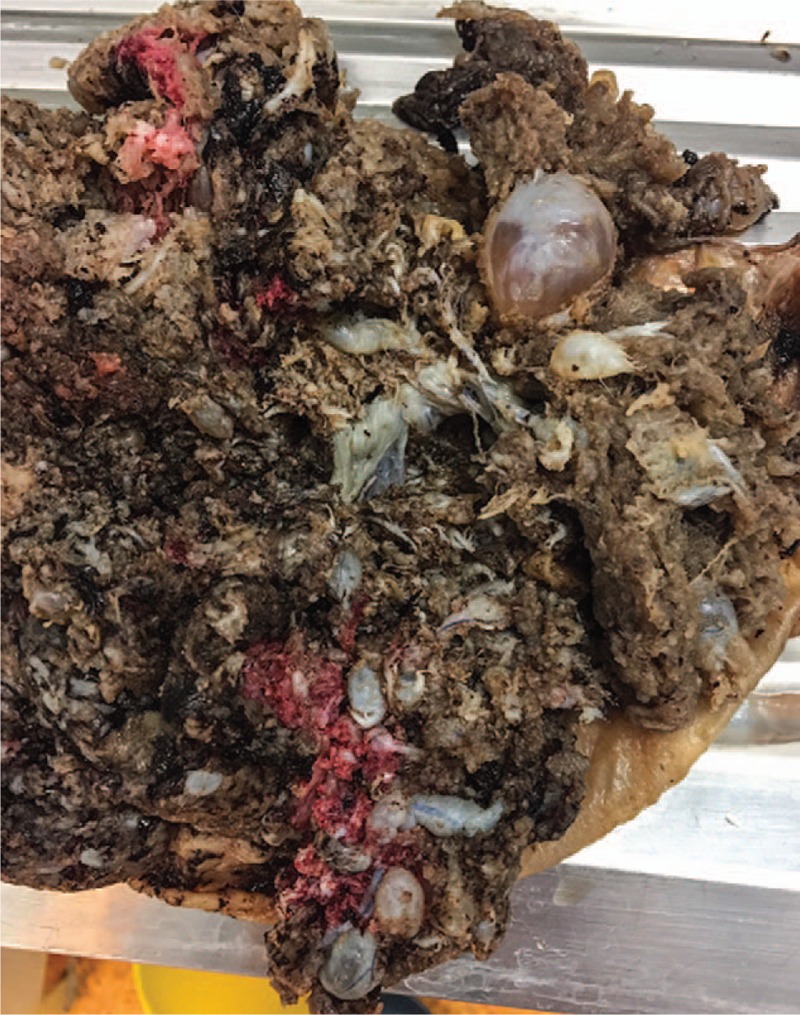
Gross image of enlarged placenta with multiple grape-like vesicles at maternal surface, especially around the cord insertion.

**Figure 4:**
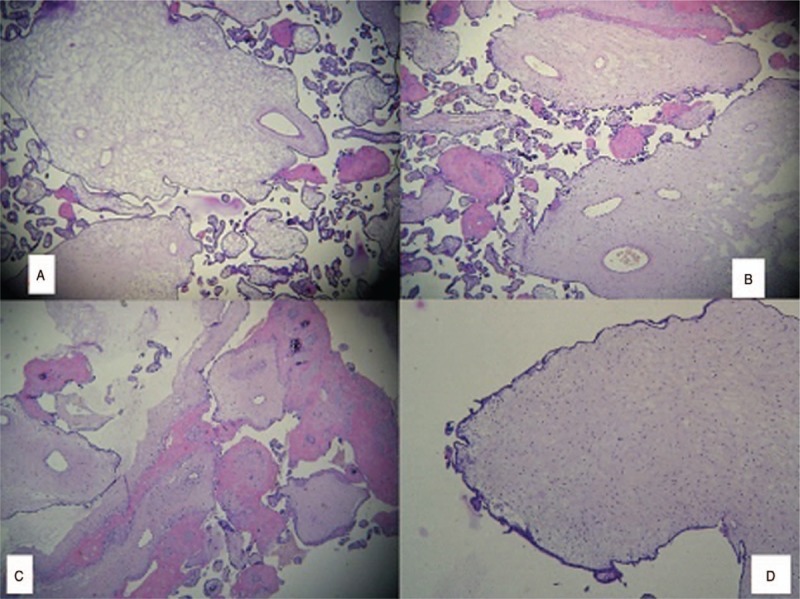
A large, edematous stem villi with central cistern formation and peripheral thick-walled vessels (40×). B, C Stem villus with myxomatous stroma and peripheral distribution of thick-walled vessels, in a mixed background of small normal and dysmature villi (40×). D, A stem villus with increased stromal mesenchymal cells (100×).

Additionally, the macroscopic evaluation showed that the placenta was markedly enlarged, weighting 680 g and measuring 14 × 15 × 4 cm. The umbilical cord was 58 cm long and 1 cm in diameter with 3 vessels. The surfaces of the placenta showed an admixture of normal-looking areas and numerous clusters of grape-like fluid-filled vesicles measuring up to 2.0 cm in diameter (Fig. 3). The umbilical cord presented an eccentric insertion. Further on, 2 types of villi populations were observed microscopically, a mostly normal for the gestational age population and the second type of villi was represented by enlarged stem villi with hydropic changes and central cistern formation, with thick-walled vessels at the periphery and myxoid stroma (Fig. 4).

## 3 Discussion

Placental mesenchymal dysplasia (PMD) is a rare benign condition with unknown underlying cause. It has been some theories that PMD is a congenital malformation of the mesoderm due to mesenchymal hyperplasia in stem villi. The enlarged stem villi contain acid mucopolysaccharide, which is found in the connective tissue layers of the normal chorionic mesoderm.^[7]^ Moreover, the most common karyotype associated with PMD, according to Truc et al,^[8]^ is 46,XX in 82% of cases and 20% of cases also have BWS but, Kaiser-Rogers et al^[9]^ proposed an androgenetic/biparental mosaicism as the etiological factor. Other type of genetic alterations found in PMD are: 11p15.5, which may affect the expression of IGF-2^[5]^ and Xp22.31, which is expressed as vascular endothelial growth factor D (VEGF-D).^[10]^

The incidence of this pathology is reported to be around 0.02% of pregnancies^[3]^ with a preponderance of 3.6–4/1 female/male.^[11,12]^ The sex of the fetus is another particularity of the case.

In our case the fetus was normal (46, XY) but Cohen et al^[13]^ described 3 cases of PMD associated with fetal aneuploidy: trisomy 13 (47,XX, t(1:3) (q32;q32)+ 13), Klinefelter syndrome (47, XXY), and triploidy (69, XXX).

It is highly important to distinguish PMD from a partial mole with an abnormal triploid fetus, because this diagnosis may result in pregnancy termination while fetus from pregnancy with PMD may develop normally without severe maternal complications. The sonographic features of PMD are very similar to those of partial moles^[2]^ and usually evidenced between 17 and 20 weeks of gestation or sometimes in the third trimester of pregnancy if the progressive development of vascular malformations secondary to circulatory disorders of the dysplastic villi is slow. Sonographic findings of PMD revealed a thickened placenta with multicystic appearance without vascular flow.^[14]^ The umbilical cord anomaly may be found like single umbilical artery, excessively long cord, marked twisted cord, and abnormal insertion like eccentrically insertion which was observed in our case.

The etiology of intrauterine growth restriction comprise: infections, placental insufficiency and other type of pathology (chorioangioma, PMD), hypertension, genetic anomalies, uterine pathology, and age. In this case is very difficult to say the exact etiology of the IUGR because the patient has 4 factors for intrauterine growth restriction: age, polyfibromatosis, hypertension, and PMD. Late apparition of the growth restriction as well as PMD ultrasound images takes us to the supposition that PMD is the cause of growth restriction. The exact incidence of IUGR in pregnancies with PMD is unknown. Pham et al^[10]^ reported, in a small cohort of 11 cases, a incidence of IUGR around 50% and intrauterine fetal demise around 43%.

According to Faye-Petersen fetuses and newborns with PMD are at risk for fetomaternal hemorrhage^[15]^ that's why we suppose that in this case the intracerebral hemorrhage may be due to PMD rather than prematurity.

## 4 Conclusions

The particularity of this case is the late development of the PMD and the severe IUGR with neonatal fetal death due to inter-cerebral hemorrhage. The severe growth restriction may also be in conjunction with the advanced maternal age and associated pathology (uterine poly-fibromatosis, hypertension, and obesity grade II). Another particularity is that the fetus was male and without any genetic anomaly.

In conclusion, we would like to emphasize on the importance of evaluating the placenta at every ultrasound examination because PMD has high rates of IUGR and IUFD and it is very important to identify it prenatally in an effort to reduce fetal morbidity and mortality.

## Author contributions

**Conceptualization:** Bogdan Doroftei, Natalia Plopa, Emil Anton, Radu Maftei.

**Investigation:** Bogdan Doroftei, Gabriela Simionescu, Delia Grab, Emil Anton.

**Methodology:** Bogdan Doroftei.

**Resources:** Bogdan Doroftei.

**Supervision:** Bogdan Doroftei, Sabina Neculai-Valeanu.

**Validation:** Bogdan Doroftei.

**Visualization:** Gabriela Simionescu.

**Writing – original draft:** Bogdan Doroftei, Sabina Neculai-Valeanu, Natalia Plopa, Emil Anton, Radu Maftei.

**Writing – review & editing:** Bogdan Doroftei, Sabina Neculai-Valeanu, Gabriela Simionescu, Delia Grab, Radu Maftei.
